# Attenuated lncRNA NKILA Enhances the Secretory Function of Airway Epithelial Cells Stimulated by *Mycoplasma pneumoniae* via NF-*κ*B

**DOI:** 10.1155/2021/6656298

**Published:** 2021-03-26

**Authors:** Fengxia Zhang, Jiamin Zhang, Feng Liu, Yao Zhou, Yun Guo, Qingning Duan, Yifan Zhu, Deyu Zhao, Haiyan Gu

**Affiliations:** ^1^Department of Respiratory Medicine, Children's Hospital of Nanjing Medical University, Nanjing 210008, China; ^2^Department of Emergency Medicine, Children's Hospital of Nanjing Medical University, Nanjing 210008, China; ^3^Department of Respiratory Medicine, Wuxi Children's Hospital, Wuxi 214000, China; ^4^Department of Pediatrics, Taizhou People's Hospital, Taizhou 225700, China

## Abstract

The secretory function of airway epithelial cells is important in the pathogenesis of *Mycoplasma pneumoniae* pneumonia (MPP). To investigate the regulatory function of NKILA (nuclear factor-*κ*B (NF-*κ*B) interacting long noncoding RNA (lncRNA)) in MPP, we first detected NKILA as well as the concentration of interleukin 8 (IL-8) and tumor necrosis factor-*α* (TNF-*α*) in bronchoalveolar lavage fluid of children with MPP. Then, NKILA was knocked down in epithelial cells to investigate its effect on their secretory function. The results suggested that NKILA was downregulated in children with MPP, while IL-8 and TNF-*α* levels increased. Knockdown of NKILA in vitro promoted the inflammatory effects of *Mycoplasma pneumoniae* (MP) in epithelial A549 and BEAS-2B cells. Knockdown of NKILA promoted inhibitor of *κ*B*α* (I*κ*B*α*) phosphorylation and degradation, and NF-*κ*B p65 nuclear translocation. Furthermore, RNA immunoprecipitation showed that NKILA could physically bind to I*κ*B*α* in MP-treated A549 cells. Collectively, our data demonstrated that attenuation of NKILA enhances the effects of MP-stimulated secretory functions of epithelial cells via regulation of NF-*κ*B signaling.

## 1. Introduction

*Mycoplasma pneumoniae* (MP) is a major cause of community-acquired pneumonia, especially in children and young adults [1]. Although MP infection is typically self-limiting, more and more cases have been reported to progress to severe, refractory, and even life-threatening pneumonia [2]. Accumulating evidence demonstrated that excessive airway inflammation plays an essential role in the development of *Mycoplasma pneumoniae* pneumonia (MPP) [3–5].

Airway epithelial cells are barrier cells on the surface of the airways, which have active secretory functions. When activated by external stimuli such as infection, they release a variety of cytokines, thus playing a key role in airway inflammation [6]. Excessive production of cytokines can aggravate pulmonary injury through worsening damage to the respiratory epithelium cells characterized by loss of cilia, vacuolation, and exfoliation [7]. Cytokine secretion is a complex process, in which nuclear factor-*κ*B (NF-*κ*B), an important inflammatory signaling pathway component, plays pivotal roles [8]. According to previous research, the lipoprotein components of MP can recognize toll-like receptors and then activate NF-*κ*B to regulate the secretion of various inflammatory mediators and chemokines, thus generating airway inflammation [9]. Among multiple inflammatory cytokines, interleukin 8 (IL-8) and tumor necrosis factor-*α* (TNF-*α*) have been reported to be prominent for the clinical evaluation of MPP [10].

Long noncoding RNAs (lncRNAs) are a subtype of noncoding transcripts with more than 200 bases of length, which can regulate a variety of physiological and pathological processes [11, 12]. lncRNAs may act as enhancers or inhibitors of inflammatory transcription to regulate the dynamics and epigenetics of this process. NF-*κ*B interacting lncRNA (NKILA), which is associated with the NF-*κ*B signaling pathway, has been reported to inhibit multiple inflammatory diseases [13–15]. However, whether NKILA could act as a regulator of inflammation in MPP has not been reported to date.

In this study, we aimed to investigate the potential role of NKILA in airway inflammation of children with MPP and the effect of NKILA on the secretory function of airway epithelial cells *in vitro*.

## 2. Materials and Methods

### 2.1. Study Subjects

Children with MPP admitted to the Children's Hospital of Nanjing Medical University from September 2018 to December 2018 were enrolled in this study. Children with intrabronchial foreign bodies (FB) were included as controls. The diagnosis of FB depends on the history of airway foreign body inhalation and bronchoscopy results. The diagnosis of pneumonia is based on clinical manifestations including fever, cough, dyspnea, abnormal breathing sounds, and pulmonary imaging abnormalities. MP infection was confirmed by polymerase chain reaction (PCR) for MP in nasopharyngeal secretions and/or serologic testing. Subjects who suffered from chronic underlying conditions, heart diseases, and immune deficiencies or used immunosuppressive drugs were excluded. Besides, the subject's nasopharyngeal secretions needed to test negative for respiratory syncytial viruses, influenza viruses, parainfluenza virus, metapneumovirus, adenovirus, *Chlamydia trachomatis*, and bacterial cultures. The clinical data are shown in Table 1.

### 2.2. Bronchoscopy and Bronchoalveolar Lavage

Guidelines for bronchoscopy and alveolar lavage were reviewed in previous literatures [16, 17]. We followed the methods described in our recent publication [18]. Children with MPP were subjected to BALF collection within 1 week after admission. For children with FB, the foreign body was removed by bronchoscopy immediately after admission, and BALF specimens were collected during the reexamination of bronchial FB. Lavage was performed with sterile saline solution. BALF was then centrifuged at 1000× *g* at 4°C for 5 min within 1 h of collection; the supernatant was stored at -20°C, while the precipitate was resuspended in Trizol reagent (Invitrogen, Carlsbad, USA) and stored at -80°C.

### 2.3. Cell Culture

A549 and BEAS-2B cells (Shanghai Cellular Research Institute, Shanghai, China) were maintained in DMEM (Gibco, Grand Island, USA) with 10% fetal bovine serum (Gibco), at 37°C in a humidified 5% CO_2_ incubator.

### 2.4. Mycoplasma Culture and Infection

MP international standard strain M129 was provided by Professor Chen Z. M. (Children's Hospital, Zhejiang province, China). The strain was cultured in a *Mycoplasma* broth, which consists of *Mycoplasma* broth base (Oxoid, Basingstoke, UK), 0.5% glucose, 0.002% phenol red, and *Mycoplasma* selective supplement G (Oxoid) as we previously described [18, 19]. MP was quantified by counting the number of colony-forming units (CFU) in *Mycoplasma* agar plates [20]. For *in vitro* experiments, MP were harvested by centrifugation (10000× *g* for 20 min), washed, and resuspended in phosphate-buffered saline (PBS) to yield 1 × 10^8^ CFU/mL. Cells were incubated with MP solution (100 CFU/mL) for 18 h [21].

### 2.5. Small Interfering RNA (siRNA) and Cell Transfection

SiRNAs against NKILA and control-scrambled siRNA were designed and synthesized by Ribo Biotechnology Co., Ltd. (Guangzhou, China). Transfection of vectors was performed using Ribo FECT™ CP (Ribo Biotechnology Co., Ltd.) in accordance with the manufacturer's protocol. Sequences of custom siRNA are listed in Table 2.

### 2.6. Quantitative Real-Time PCR (qRT-PCR) Assay

Total RNA was extracted from cells using Trizol reagent according to manufacturers' instructions (Ambion, Austin, USA). Then RNA was reverse translated to complementary DNA using the PCR Master Mix (Vazyme Biotech, Nanjing, China). qRT-PCR was performed using an AceR qPCR SYBR Green Master Mix (Vazyme Biotech) according to the manufacturer's instructions. The expression of NKILA was normalized to glyceraldehyde 3-phosphate dehydrogenase (GAPDH) RNA expression and calculated using the 2^−*ΔΔ*Ct^ method. The primers used for qRT-PCR are listed in Table 3.

### 2.7. ELISA

The cytokines in the cell culture supernatant or BALF of children with MPP or FB were analyzed. The concentrations of IL-8 and TNF-*α* were detected using Human IL-8 ELISA Kit (Novus Biologicals, Littleton, USA) and Human TNF-*α* ELISA Kit (BD Pharmingen, San Diego, USA), respectively, according to the manufacturer's instructions.

### 2.8. Western Blot

Cells were washed twice with ice-cold PBS, and then proteins were extracted with the Total Protein Extraction Kit or nuclear cytoplasm extraction kit (KeyGen Biotech, Nanjing, China). Aliquots of 30 *μ*g lysed proteins of each group were separated by sodium dodecyl sulfate polyacrylamide gel electrophoresis and then transferred to polyvinylidene difluoride membranes (Merck Millipore, Billerica, USA). After blocking with 5% skim milk, the membranes were incubated with the primary antibody at 4°C overnight and then incubated with a secondary antibody at room temperature for 1 h. Finally, chemiluminescent detection was performed using ECL reagent (Beyotime Institute of Biotechnology, Nantong, China). Primary antibodies against p65, phosphorylated p65, inhibitor of *κ*B*α* (I*κ*B*α*), and phosphorylated I*κ*B*α* (Cell Signaling Technology, Danvers, USA) and GAPDH, *β*-actin, and histone H3 (Proteintech Group, Chicago, USA) as well as secondary goat anti-rabbit or anti-mouse antibodies (Proteintech Group) were used for immunoblot analysis.

### 2.9. Luciferase Reporter Assay

NF-*κ*B transcriptional activity reporter plasmid pNF*κ*B-luc (Beyotime Institute of Biotechnology) and Renilla luciferase expression vector pRL-TK (Promega, Madison, USA) were cotransfected into normal or NKILA depleted A549 and BEAS-2B cells using Lipofectamine 2000 (Invitrogen, Carlsbad, USA). After 24 h of transfection, cells were treated with or without MP and harvested 18 h later [18, 22]. The luciferase activity was measured using the Dual-Luciferase Reporter Assay System (Promega) in accordance with the manufacturer's protocol. The relative Firefly luciferase activity was normalized to Renilla luciferase activity.

### 2.10. RNA Immunoprecipitation (RIP)

Magna RIP RNA-Binding Protein Immunoprecipitation Kit (Merck Millipore) was used for RIP according to the manufacturer's instructions. Normal rabbit IgG (Merck Millipore) or anti-p65/I*κ*B*α* antibodies (Santa Cruz, Dallas, USA) were used for immunoprecipitation. Coprecipitated RNAs were quantified and analyzed by qRT-PCR.

### 2.11. Statistical Analysis

Each experiment was performed in triplicate, and data were represented as the mean ± standard deviation. All statistical analyses were conducted by SPSS software, version 19.0 (IBM, Armonk, USA). Comparisons of groups were analyzed using Student's *t* test while multiple comparisons using a one-way analysis of variance (ANOVA) test. The Mann-Whitney test was used to compare the abnormal distributional variables between two groups. *P* < 0.05 was defined as statistically significant.

## 3. Results

### 3.1. NKILA Was Downregulated in the BALF of Children with MPP

**Figure 1:**
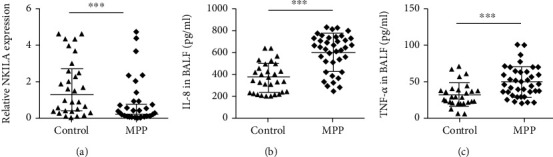
The expression of NKILA and inflammatory cytokines in BALF. (a) NKILA was downregulated in BALF of 38 children with MPP compared with 30 children with FB. The expression of NKILA was measured by qRT-PCR and normalized to GAPDH. Results are shown as the median with an interquartile range. (^∗∗∗^*P* < 0.001 by the Mann-Whitney test). (b, c) Levels of cytokines (IL-8 and TNF-*α*) in BALF of children with MPP were much higher than those in BALF of children with FB. Data are shown as the mean ± standard deviation (SD) from three experiments (^∗∗∗^*P* < 0.001).

First, we detected NKILA in the BALF of 38 hospitalized children with MPP and 30 children with FB using qRT-PCR. The results demonstrated that NKILA mRNA was significantly decreased in BALF of the MPP group compared to the ones of the control group (Figure 1(a)). To explore the regulatory role of NF-*κ*B on the secretion of airway inflammatory cytokines in MPP, we detected the concentrations of IL-8 and TNF-*α* in the BALF of both groups. As shown in Figures 1(b) and 1(c), the levels of IL-8 and TNF-*α* in BALF were significantly higher in the MPP group compared to the control group. These results demonstrated that NKILA was downregulated in the BALF of children with MPP, accompanied by an increase of the inflammatory cytokines IL-8 and TNF-*α*.

### 3.2. The Secretion Function Was Enhanced after Knockdown of NKILA in MP-Stimulated Airway Epithelial Cells

**Figure 2:**
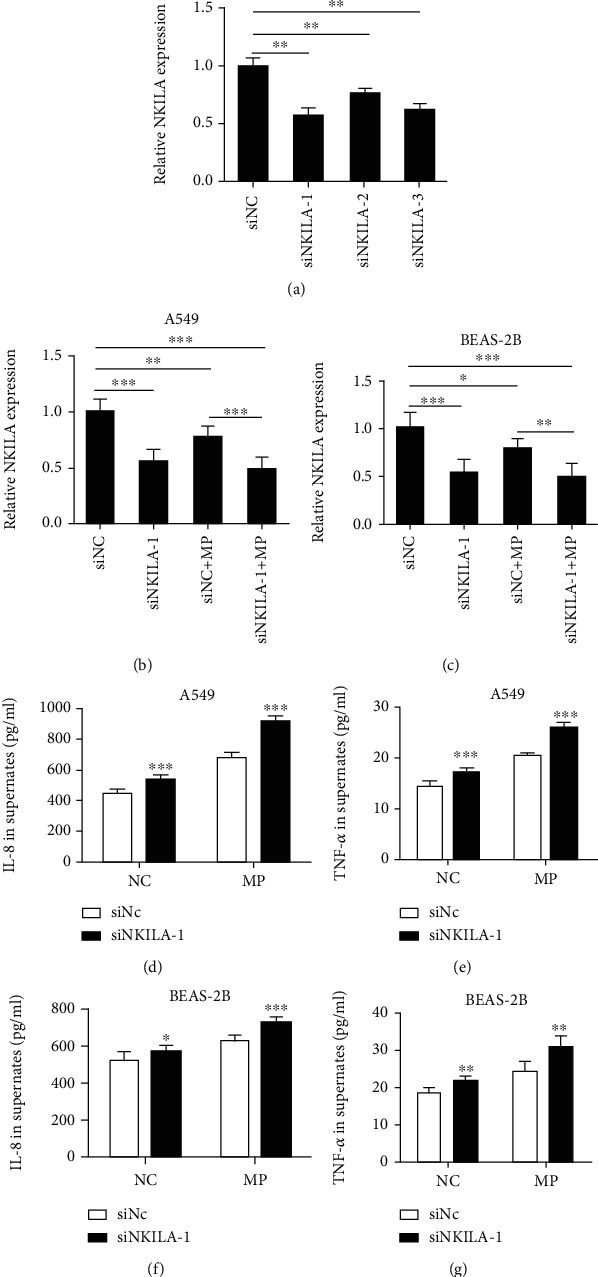
The secretion function was enhanced after knockdown of NKILA in MP-treated airway epithelial cells. (a) NKILA was significantly downregulated by the three siRNAs. (b, c) The expression of NKILA was downregulated in MP-treated A549 and BEAS-2B cells, especially in the NKILA knockdown group. (d–f) The levels of IL-8 and TNF-*α* in the supernatant of A549 and BEAS-2B cells were elevated after MP infection, while knocking down NKILA enhanced this effect. Data are shown as the mean ± SD from three experiments (^∗∗∗^*P* < 0.001, ^∗∗^*P* < 0.01, and ^∗^*P* < 0.05).

To investigate the possible biological function of NKILA in epithelial cells infected by MP, siRNA was used to silence NKILA. Three siRNA sequences against NKILA were transfected into A549 cells. As shown in Figure 2(a), NKILA was significantly downregulated by the three siRNAs, among which siNKILA-1 showed the highest efficiency. Therefore, siNKILA-1 was used in subsequent transfection experiments. Twenty-four hours after transfection, cells were stimulated with M129 for 18 h. The expression of NKILA was detected by qRT-PCR, and the results are shown in Figures 2(b) and 2(c). NKILA was downregulated in MP-treated A549 and BEAS-2B cells, especially in the NKILA knockdown group. Using ELISA, we detected the levels of inflammatory cytokines (IL-8 and TNF-*α*) in the supernatant of NKILA-depleted or normal A549 and BEAS-2B cells with or without MP treatment. As shown in Figures 2(d)–2(g), knocking down NKILA increased the level of IL-8 and TNF-*α* in the cell supernatant, even after treatment with MP. These results demonstrated that NKILA negatively regulates epithelial cells to secrete the inflammatory cytokines induced by MP.

### 3.3. NKILA Knockdown Promoted I*κ*B*α* Phosphorylation and p65 Translocation in Epithelial Cells

**Figure 3:**
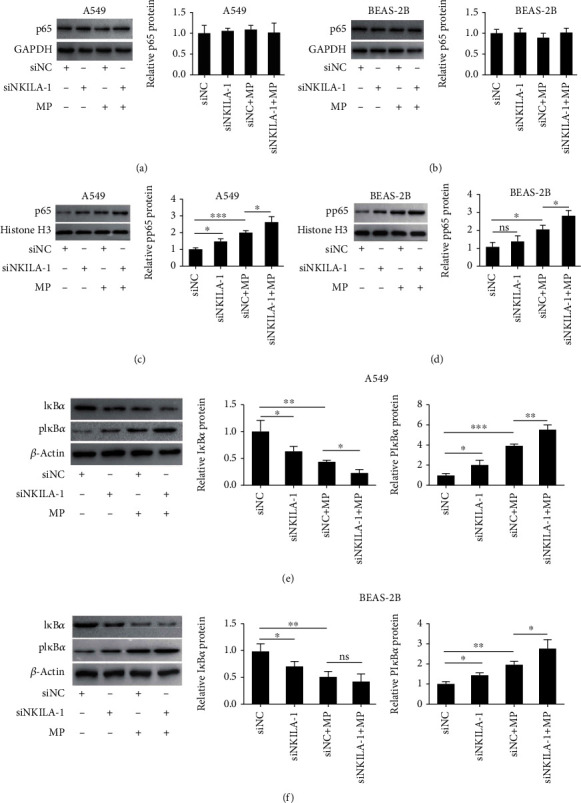
NKILA knockdown promoted I*κ*B*α* phosphorylation and p65 translocation in epithelial cells. (a, b) Total p65 protein level in NKILA-depleted and control A549 and BEAS-2B cells before and after MP infection was measured by western blot and normalized to GAPDH. (c, d) Nuclear p65 phosphorylation level was measured by western blot and histone H3 was the loading control. (e, f) Cytoplasmic I*κ*B*α* and I*κ*B*α* phosphorylation levels in NKILA-depleted and control A549 and BEAS-2B cells with or without MP infection were measured by western blot and normalized to *β*-actin. The samples were derived from the same experiment and gels/blots were processed in parallel. Data are shown as the mean ± SD from three independent experiments (^∗∗∗^*P* < 0.001, ^∗∗^*P* < 0.01, and ^∗^*P* < 0.05).

To study the relationship between NKILA downregulation and NF-*κ*B activation in MP-treated A549 and BEAS-2B cells, siNKILA-1 was utilized to knockdown NKILA and MP treatment was performed. We observed by western blot that the total amount of p65 in total protein extracts of A549 and BEAS-2B cells after NKILA silencing did not change (Figures 3(a) and 3(b)); however, the level of phosphorylated p65 in the cell nucleus extracts was significantly increased (Figures 3(c) and 3(d)). In addition, the level of phosphorylated I*κ*B*α* was increased in the cytoplasm of NKILA-depleted epithelial cells after MP infection, while the expression of I*κ*B*α* was decreased (Figures 3(e) and 3(f)). Taken together, these results demonstrated that NKILA knockdown promoted I*κ*B*α* phosphorylation and degradation and p65 translocation in MP-treated airway epithelial cells.

### 3.4. NKILA Inhibits NF-*κ*B Activity by Physically Binding to I*κ*B*α*

**Figure 4:**
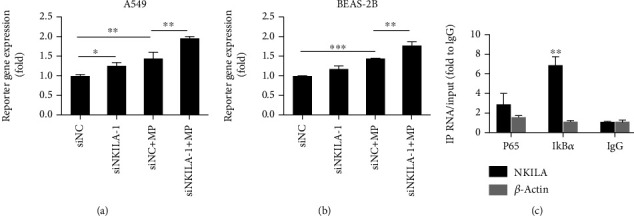
NKILA inhibits NF-*κ*B activity by binding to I*κ*B*α*. (a, b) NF-*κ*B activity of A549 and BEAS-2B cells was detected using a dual-luciferase reporter assay. (c) Interaction between NKILA and p65/I*κ*B*α* revealed by RIP experiments. Cell extracts of MP-stimulated A549 cells were immunoprecipitated with control IgG or anti-p65/I*κ*B*α* antibody, and the complexes were analyzed for the presence of *β*-actin mRNA and NKILA by qPCR. Data are shown as the mean ± SD from three independent experiments (^∗∗∗^*P* < 0.001, ^∗∗^*P* < 0.01, and ^∗^*P* < 0.05).

The effects of NKILA on NF-*κ*B activity were examined by the dual-luciferase reporter assay. The results showed that MP treatment increased NF-*κ*B transcriptional activity, while knockdown of NKILA further increased NF-*κ*B activity (Figures 4(a) and 4(b)). To prove that NKILA could physically bind to NF-*κ*B and thereby inhibit its activation, we performed RIP for cell extracts of MP-stimulated A549 cells using the p65 and I*κ*B*α* antibodies. We observed roughly sevenfold enrichment of I*κ*B*α* in the immunoprecipitates compared to the IgG control (Figure 4(c)), indicating that NKILA physically associates with I*κ*B*α*. Based on these results, we believe that NKILA binds to I*κ*B*α*, thus preventing phosphorylation of I*κ*B*α* and NF-*κ*B activation in MP-infected airway epithelial cells.

## 4. Discussion

IL-8 and TNF-*α*, known to be two important proinflammatory cytokines, play key roles in inflammation and chemotaxis in the airways caused by MP. Airway epithelium is the main source of IL-8, and lung pathogenesis is known to be correlated with increased IL-8 levels [23]. Besides, elevated TNF-*α* causes an excessive inflammatory response and induces the release of other inflammatory factors, leading to the damage of cells, tissues, and even multiple organs and systems. In the present study, we focused on the role of NKILA in regulating airway epithelial secretory functions in MPP. Our results showed that NKILA expression was downregulated in BALF of children with MPP; however, the levels of IL-8 and TNF-*α* were significantly increased in children with MPP compared to children with FB. Therefore, it was speculated that NKILA plays a potential role in regulating airway inflammation in MPP.

By conducting *in vitro* experiments, we showed that MP stimulation upregulated IL-8 and TNF-*α* in epithelial A549 and BEAS-2B cells. In order to explore the regulatory role of NKILA in the secretion of cytokines, we knocked down NKILA in epithelial cells and found that silencing of NKILA further increased the levels of proinflammatory cytokines secreted by A549 and BEAS-2B cells treated with MP. Therefore, we can conclude that NKILA plays a negative regulatory role in the inflammation produced by epithelial cells and caused by MP.

NF-*κ*B, an important transcription factor involved in the regulation of various inflammatory response genes, is known to play an important role in airway inflammation caused by MP infection. Under physiologic conditions, NF-*κ*B binds to an inhibitor protein named I*κ*B and is retained in the cytoplasm. Upon stimulation, I*κ*B is phosphorylated and degraded and then separated from NF-*κ*B. Further, the phosphorylated NF-*κ*B p65 translocates into the nucleus and activates the transcriptional expression of downstream genes associated with inflammation [24]. According to previous studies, NKILA can block I*κ*B degradation by masking the phosphorylation motifs of I*κ*B and hinder NF-*κ*B translocation [13, 15, 25, 26], thus inhibiting the transcription of downstream inflammatory response genes [27].

In the current study, we observed that MP stimulation induced NF-*κ*B activation in A549 and BEAS-2B cells. Knocking down NKILA facilitated I*κ*B*α* phosphorylation and degradation and p65 translocation, thus triggering NF-*κ*B activation. Our findings on the regulatory effect of NKILA on NF-*κ*B are consistent with the previous studies [28–30]. Additionally, in MP-stimulated A549 cells, we confirmed that NKILA physically associated with I*κ*B*α*. Liu et al. reported that NKILA physically associates with NF-*κ*B/I*κ*B and blocks I*κ*B phosphorylation, thus inhibiting NF-*κ*B activation in breast cancer cells [13]. However, we did not detect its binding to NF-*κ*B p65. The use of different cell lines and treatment methods could explain these inconsistent results. However, our results suggested that NKILA could negatively regulate NF-*κ*B activation by directly binding to I*κ*B*α* in MP-treated epithelial cells.

To sum up, we demonstrated that NKILA was downregulated in BALF of children with MPP, while the levels of IL-8 and TNF-*α* increased. Knockdown of NKILA promoted epithelial cells to secrete MP-induced cytokines. The effects of NKILA on the epithelium were achieved by negative regulation of NF-*κ*B. Therefore, we conclude that attenuating lncRNA NKILA enhances the secretory function of airway epithelial cells stimulated by MP via regulation of NF-*κ*B signaling. Thus, NKILA may serve as a critical therapeutic target for regulating the excessive airway epithelial inflammation caused by MP infection. Our study provides a new theoretical basis for further revealing the inflammatory mechanism of MPP and a potential intervention target for blocking the progression of MPP, which might bring a new perspective to improve the prognosis of children with severe MPP.

## 5. Conclusion

Our study revealed that NKILA can affect the secretion function of epithelial cells induced by MP infection via regulation of NF-*κ*B signaling. This finding provides new insights into the molecular mechanisms of inflammation in MPP. NKILA may be a potential therapeutic target for MPP.

## Figures and Tables

**Table 1:** Comparison of general data between two groups.

	MPP group (*n* = 38)	FB group (*n* = 30)	*P* value
Age (years)	3.015 ± 1.283	2.471 ± 1.231	0.082
Sex (male/female)	21/17	19/11	0.502

**Table 2:** Sequences of custom siRNAs.

	Sequences
siNC	5′-UUCUCCGAACGUGUCACGU-3′
siNKILA-1	5′-GCCAGAAACTCTCCAAATA-3′
siNKILA-2	5′-CAGGAGTGCTACAAGAACA-3′
siNKILA-3	5′-CGCTGCAACTTAAGAGAAA-3′

siNC: control-scrambled siRNA; siNKILA1-3: siRNAs against NKILA.

**Table 3:** Primers for qRT-PCR.

	Primers
NKILA-F	5′-AACCAAACCTACCCACAACG-3′
NKILA-R	5′-ACCACTAAGTCAATCCCAGGTG-3′
GAPDH-F	5′-GCTCTCTGCTCCTCCTGTTC-3′
GAPDH-R	5′-ACGACCAAATCCGTTGACTC-3′
*β*-Actin-F	5′-AAAGACCTGTACGCCAACAC-3′
*β*-Actin-R	5′-GTCATACTCCTGCTTGCTGAT-3′

NKILA: NF-*κ*B interacting lncRNA; F: forward; R: reverse.

## Data Availability

The data that support the findings of this study are available from the corresponding author upon request.

## Abbreviations

MPP: *Mycoplasma pneumoniae* pneumonia
MP: *Mycoplasma pneumoniae*
lncRNAs: Long noncoding RNAs
NF-*κ*B: Nuclear factor-*κ*B
NKILA: NF-*κ*B interacting lncRNA
IL-8: Interleukin 8
TNF-*α*: Tumor necrosis factor-*α*.
